# Cortical Anoxic Spreading Depolarization During Cardiac Arrest is Associated with Remote Effects on Peripheral Blood Pressure and Postresuscitation Neurological Outcome

**DOI:** 10.1007/s12028-022-01530-2

**Published:** 2022-06-21

**Authors:** Sangwoo Han, Mayra Isabel Contreras, Afsheen Bazrafkan, Masih Rafi, Shirin M. Dara, Ani Orujyan, Anais Panossian, Christian Crouzet, Beth Lopour, Bernard Choi, Robert H. Wilson, Yama Akbari

**Affiliations:** 1 Department of Neurology, University of California, Irvine, Irvine, CA USA; 2 Department of Anatomy and Neurobiology, University of California, Irvine, Irvine, CA USA; 3 Department of Biomedical Engineering, University of California, Irvine, Irvine, CA USA; 4 Beckman Laser Institute and Medical Clinic, University of California, Irvine, Irvine, CA USA; 5 Department of Surgery, University of California, Irvine, Irvine, CA USA; 6 Department of Medicine, University of California, Irvine, Irvine, CA USA

**Keywords:** Cortical spreading depolarization, Anoxic spreading depolarization, Spreading ischemia, Neurovascular coupling, Cardiac arrest, Peripheral hemodynamics, Cerebral hemodynamics

## Abstract

**Background:**

Spreading depolarizations (SDs) are self-propagating waves of neuronal and glial depolarizations often seen in neurological conditions in both humans and animal models. Because SD is thought to worsen neurological injury, the role of SD in a variety of cerebral insults has garnered significant investigation. Anoxic SD is a type of SD that occurs because of anoxia or asphyxia. Although asphyxia leading to a severe drop in blood pressure may affect cerebral hemodynamics and is widely known to cause anoxic SD, the effect of anoxic SD on peripheral blood pressure in the extremities has not been investigated. This relationship is especially important to understand for conditions such as circulatory shock and cardiac arrest that directly affect both peripheral and cerebral perfusion in addition to producing anoxic SD in the brain.

**Methods:**

In this study, we used a rat model of asphyxial cardiac arrest to investigate the role of anoxic SD on cerebral hemodynamics and metabolism, peripheral blood pressure, and the relationship between these variables in 8- to 12-week-old male rats. We incorporated a multimodal monitoring platform measuring cortical direct current simultaneously with optical imaging.

**Results:**

We found that during anoxic SD, there is decoupling of peripheral blood pressure from cerebral blood flow and metabolism. We also observed that anoxic SD may modify cerebrovascular resistance. Furthermore, shorter time difference between anoxic SDs measured at different locations in the same rat was associated with better neurological outcome on the basis of the recovery of electrocorticography activity (bursting) immediately post resuscitation and the neurological deficit scale score 24 h post resuscitation.

**Conclusions:**

To our knowledge, this is the first study to quantify the relationship between peripheral blood pressure, cerebral hemodynamics and metabolism, and neurological outcome in anoxic SD. These results indicate that the characteristics of SD may not be limited to cerebral hemodynamics and metabolism but rather may also encompass changes in peripheral blood flow, possibly through a brain–heart connection, providing new insights into the role of anoxic SD in global ischemia and recovery.

## Introduction

Cortical spreading depolarizations (SDs) are self-propagating waves of transient or persistent [1] loss of neuronal transmembrane ion gradients leading to neuronal and glial depolarizations [2]. SD is an extremely energy-demanding process and has been shown to double the cerebral metabolic rate of oxygen consumption (CMRO_2_) [2] and cause neurovascular decoupling [2–4]. SDs can occur during ischemia and in human pathological conditions, such as stroke [5] and traumatic brain injury [6, 7]. SDs in these conditions are believed to exacerbate injury by diverting blood flow and worsening ischemia [8]. Except at the ischemic core, the SDs produced in these conditions are transient, meaning repolarization occurs spontaneously [1]. In contrast, anoxic SD, a type of SD caused by anoxia or asphyxia, is persistent until return of blood flow or oxygen, after which repolarization may occur [1]. Anoxic SD is also seen in global ischemia produced by cardiac arrest and has been recently found by our laboratory to be related to outcome [9]. Specifically, in that study, an earlier anoxic SD during cardiac arrest was correlated with better outcome post resuscitation [9, 10]. Therefore, anoxic SD may be of widespread clinical importance and interest.

Although the effects of anoxic SD on cerebral hemodynamics have long been of interest, its effects on peripheral hemodynamics during brain injury are poorly understood. The potential effects of anoxic SD on both peripheral and cerebral hemodynamics has garnered more interest because recent studies have shown that the vagus nerve, which controls cardiac activity, can also modulate SD [11–14]. This brain–heart coupling via the vagus nerve raises the possibility that anoxic SD may be able to affect the heart through the vagus nerve. Currently, it is difficult in critical care settings to continuously monitor cerebral blood flow (CBF) and brain metabolism in parallel with peripheral perfusion, especially with the high temporal resolution required to quantify the dynamics of anoxic SD [9, 15]. Furthermore, it is difficult to predict the timing of anoxic injury and begin invasive monitoring procedures in advance. Therefore, investigations using animal models are necessary. With our custom-built multimodal monitoring devices in our rodent model of cardiac arrest and resuscitation, we were able to continuously monitor direct current (DC) electrocorticography, CBF, brain oxygenation, CMRO_2_, and peripheral blood pressure all simultaneously during anoxic SD.

Most studies investigate SD’s effect on CBF [2, 3, 16, 17] or CMRO_2_ [2, 4], but almost no studies have compared the changes in CBF and peripheral blood pressure and their coupling during anoxic SD. To our knowledge, only one study has been conducted on the effects of anoxic SD during cardiac arrest on peripheral hemodynamics and its coupling with cerebral hemodynamics [18]. The authors showed no changes in peripheral or cerebral hemodynamics during anoxic SD, but the time resolution they used for analysis was too low to capture rapid changes because they only used averaged signals at seven selected time points over 3 min [18]. The most extensive investigations into SD in rodent models have been in nonpathological states [4, 17, 19–21], followed by conditions known to produce SD, such as stroke, hemorrhage, and traumatic brain injury [7, 8, 18, 22, 23], rather than anoxic SD. Global ischemia, often caused by cardiac arrest, has been shown to produce anoxic SD, but few studies have investigated SD in this setting or characterized its effects on cerebral and peripheral hemodynamics [9, 15, 18]. Thus, the effects of anoxic SD on peripheral hemodynamics are not well understood. Interestingly, in a prior study, we found that another measure of cortical activity during near-electrocerebral silence of impending cardiac arrest, namely phase coherence as measured by electrocorticography, coincided with a transient rise in peripheral blood pressure, suggesting neurovascular coupling during cardiac arrest [24]. However, it is unclear if anoxic SD, which occurs well after such phase coherence in our cardiac arrest model, shares such changes in peripheral hemodynamics. It is important to investigate what hemodynamic effects anoxic SD produces in a state of global ischemia and the ramifications of these effects so that we can better understand what is happening clinically in human patients with similar conditions. In this pilot study, we aimed to investigate the effects of anoxic SD on peripheral and cerebral hemodynamics and outcome. In particular, in our cardiac arrest model of global ischemia, we found that blood flow to the brain shuts down, leading to anoxic SD throughout the brain. We observed that anoxic SD onset occurred at different times at different electrodes. On the basis of this observation as well as previous literature [25], we hypothesized that our observed anoxic SD is multifocal and the time delay between the anoxic SD onsets at different foci may be a signature of a broad connectivity-related metric while the brain shuts down during anoxia. We measured the time delay between anoxic SD onsets at two different electrodes, which we termed the “∆SD1-2 period” to represent asynchronous depolarizations of different brain regions. We saw fluctuations in peripheral (femoral) blood pressure and CBF during this period. On the basis of our recent studies showing that post-cardiac-arrest outcome is related to coupling of the neurovascular unit as well as dissociation between features of peripheral blood pressure and CBF [41, 43], we hypothesized that peripheral perfusion and CBF change during the ∆SD1-2 period may also affect outcome. We found that a shorter ∆SD1-2 period and a faster drop in peripheral perfusion during this period may be associated with better neurological outcome. This suggests that the consequences of anoxic SD stretch further than just within the brain and may impact the overall pathological process and even potentially recovery in conditions such as cardiac arrest.

## Methods

In this study, we analyzed a naturally occurring anoxic SD during asphyxial cardiac arrest using Ag/AgCl DC electrodes, optical imaging, and femoral artery cannulation for peripheral blood pressure monitoring at a high temporal resolution. For optical imaging, we used laser speckle imaging to monitor CBF and multispectral spatial frequency domain imaging to monitor CMRO_2_. For the purpose of simplicity in the Methods and Results sections, where only anoxic SD is mentioned, we will refer to anoxic SD as just SD.

### Preclinical Rodent Model of Asphyxial Cardiac Arrest: Surgical Preparation

**Fig. 1:**
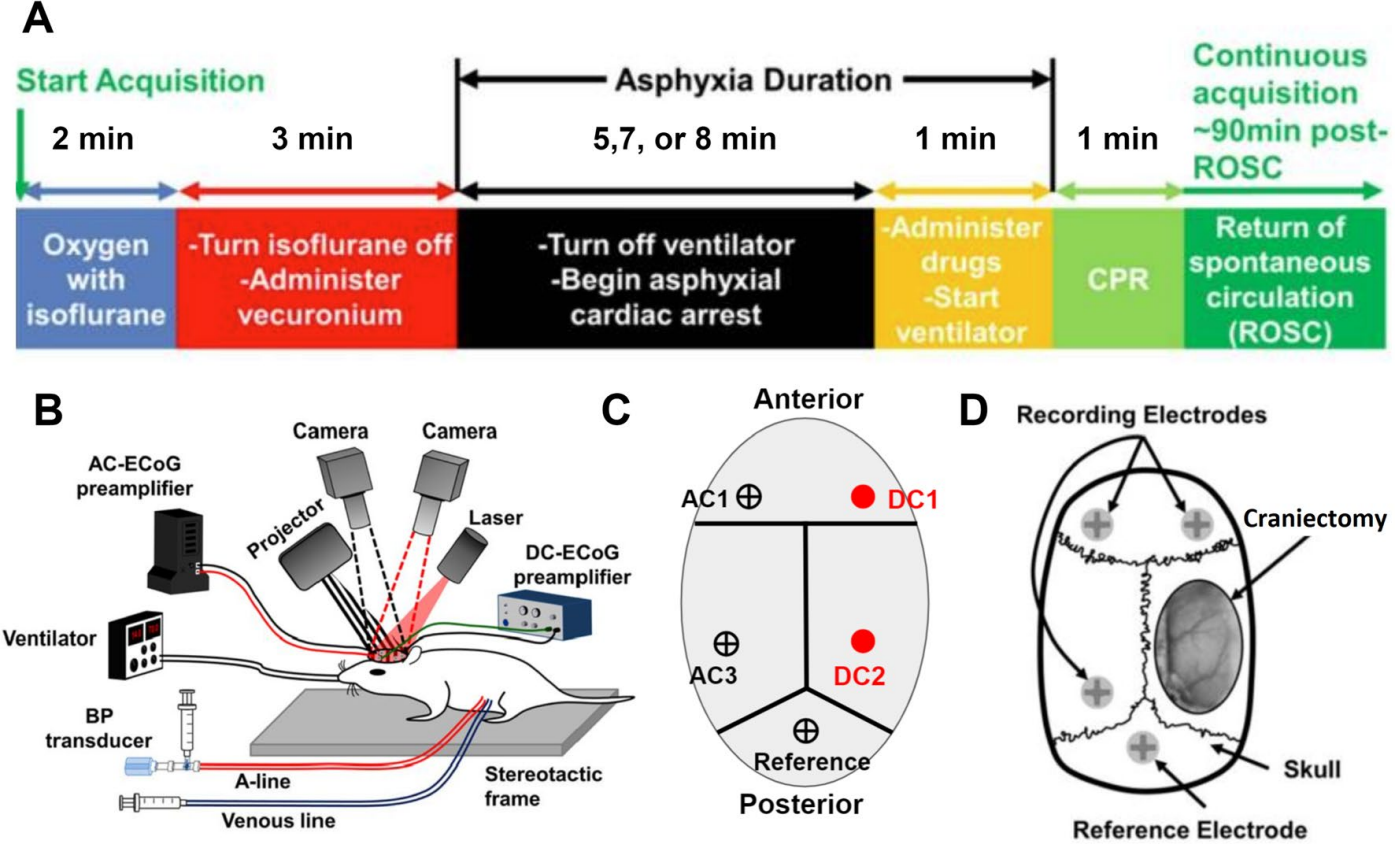
Experimental timeline and set up. **a** Timeline of the asphyxial cardiac arrest experiment. **b** Schematic of our multimodal monitoring platform during the experiment. **c** Location of the AC and DC electrodes for cohort 1. **d** Location of the AC electrodes and craniectomy window for cohort 2. Reprinted/adapted with permission from Crouzet et al. [15] © The Optical Society

The animal model of asphyxial cardiac arrest and resuscitation used in this study has been described previously [9, 26–28] by our laboratory and is summarized in Fig. 1. Briefly, male Wistar rats (Charles River Laboratories) of 8 to 12 weeks of age weighing 300 to 400 g were handled daily for at least 1 week prior to any experimentation. The night prior to cardiac arrest experiments, rats were calorically restricted (75% restriction) with three food pellets, as is standard with our model [26]. The next morning, rats were endotracheally intubated and received femoral artery and vein cannulation to enable arterial blood pressure monitoring and venous drug delivery, respectively. The temperature of the animals was maintained at 37 °C using a temperature probe and heating pad.

### Preclinical Rodent Model of Asphyxial Cardiac Arrest: Cardiac Arrest

Before the experiment (Fig. 1a), the rats were on 50% oxygen, 50% nitrogen; this was subsequently switched to 100% oxygen at the beginning of the experiment. An anesthetic (isoflurane) was washed out over a 3-min period, and a paralytic agent (vecuronium) was given. Following the washout period, cardiac arrest was induced by turning off the ventilator for 8 min. Subsequently, cardiopulmonary resuscitation was initiated by turning the ventilator back on with 100% oxygen, giving epinephrine and sodium bicarbonate, and performing manual chest compressions. Chest compressions were continued until the return of spontaneous circulation, after which the rats were monitored continuously for an additional 2 to 4 h. When the rat was able to breathe on its own, it was taken off the ventilator and breathed room air.

### Preclinical Rodent Model of Asphyxial Cardiac Arrest: Postresuscitation Assessment

Staff assessing neurological outcome were blinded to the experimental groups. The neurological deficit scale, a behavioral test, was used to score neurological function at 24 h post resuscitation, with a higher score representing better neurological function [29–31]. The burst suppression ratio, a quantification of suppression of alternating current (AC)-ECoG activity, was also used as an outcome measure at 40 to 60 min post resuscitation, when the rat was neurologically recovering. The burst suppression ratio decreases as the rat regains consciousness. Time to burst is the time to resumption of electrical activity of the brain as measured by the first burst of activity in the AC-ECoG signal and was used as another marker of neurological recovery.

### Experimental Groups

In this study, two separate cohorts of rats, each with different multimodal monitoring techniques, were used (Fig. 1b). Animals were randomized into these two cohorts. In cohort 1 (Fig. 1c), 14 rats underwent surgery to implant three screw electrodes for AC-ECoG recordings and two DC Ag/AgCl electrodes for DC-ECoG recordings 1 week prior to the asphyxial cardiac arrest experiment. All rats in this cohort underwent an 8-min asphyxial cardiac arrest. The Ag/AgCl electrodes were made by chloridizing silver wires (Stoelting 50,880) in household bleach. The Ag/AgCl electrodes had a diameter of 0.01 inches and were implanted 1 mm into the cortex in the right frontal hemisphere; another one was implanted in the right parietal cortex. Implanting and recording at the level of the cortex has a better signal-to-noise ratio, has higher spatial and temporal resolution, and is less prone to artifacts [32]. We did not find any histological signs of neurodegeneration in the implantation sites. The skin at the back of the neck for each rat was clamped with an alligator clip to serve as a reference for the DC electrodes. Ten rats in cohort 2 (Fig. 1d) were monitored with optical imaging after we performed a partial craniectomy over the right parietal lobe with the dura intact (not removed) on the day of the experiment. Rats in this cohort were monitored continuously with AC-ECoG electrodes but did not have DC-ECoG monitoring. These animals did not survive owing to the craniectomy. DC electrodes were used to monitor SD in cohort 1, and optical scattering changes were used to monitor SD in cohort 2. For analysis of outcome in cohort 1, two rats were excluded from the analysis as statistical outliers because they had significantly higher post-cardiac-arrest blood glucose levels, which could have been due to additional surgical stress. However, these two rats were included in the analyses not pertaining to outcome because only time periods before resuscitation were analyzed in those cases.

### Data Acquisition and Post Processing

Post processing of arterial blood pressure, AC-ECoG, DC-ECoG, and optical imaging data was performed using MATLAB (The MathWorks, Inc., Natick, MA). In this study, AC-ECoG data were primarily used for the analysis of ECoG burst onset and the burst suppression ratio.

### Burst Suppression Ratio

To calculate the burst suppression ratio for each rat, the AC-ECoG signal was processed by down sampling by 6 to 254 Hz to reduce computational load, performing common average referencing for two AC-ECoG channels (AC1, AC3), notch 60-Hz filtering to remove electrical noise, and 1- to 150-Hz bandpass filtering. Afterward, a threshold for the amplitude of the signal was calculated by taking the sum of the mean and standard deviation during a period of 20 to 30 min post resuscitation. A burst suppression was calculated at an interval of at least 0.5 s, in which the amplitude of the AC-ECoG signal did not exceed the threshold. The ratio of the time in burst suppression was calculated over a period of 1 min with an overlapping window of 30 s. This was calculated from 5 min prior to the onset of asphyxia to 100 min after. For this study, we used an average of the burst suppression ratio during the 40- to 60-min period after onset of asphyxia as one of the markers for neurological outcome.

### Time to Burst

Time to burst was obtained by visually observing the first burst of AC-ECoG activity on our livestream of the AC-ECoG signal post resuscitation and is another marker of recovery in animals waking from coma after cardiac arrest. This was double checked by plotting the AC-ECoG signal with MATLAB and confirming the first burst of activity.

### Arterial Blood Pressure and Arterial Blood Gas

For both cohorts, blood pressure was measured continuously at 191 Hz from an arterial line. Mean arterial pressure (MAP) was calculated using the following formula: MAP = (systolic blood pressure + [2 × diastolic blood pressure])/3.

### DC-ECoG

In cohort 1, DC-ECoG data were acquired at 305 Hz with Duo 773 and Model 750 electrometers (World Precision Instruments, Saratosa, FL) with two DC leads, DC1 and DC2. A 1-Hz low pass filter was used on the DC-ECoG signal post acquisition to remove noise and identify SD. DC1 (located in the right frontal cortex) and DC2 (located in the right parietal cortex) both produced SD at different times, shown as DC potential shifts. SD was detected with DC electrodes by observing a DC potential shift and visually identifying the negative deflection in DC-ECoG to mark the SD onset.

### Optical Imaging

Throughout the experiments, rats in cohort 2 were monitored with two diffuse optical imaging technologies: laser speckle imaging and multispectral spatial frequency domain imaging. Laser speckle imaging measures blood flow, as previously established in both preclinical and clinical studies [33–35], including investigations into cardiac arrest [28, 33, 36]. Spatial frequency domain imaging [37, 38] measures the tissue absorption coefficient (related to hemoglobin concentration and oxygenation [28, 37, 39]) and scattering coefficient (related to morphology and distribution of cells and organelles [40]). Tissue scattering has been shown to change during SD, likely because of cytotoxic edema [39]. The combination of laser speckle imaging and spatial frequency domain imaging data can be used to calculate CMRO_2_ [41]. In this study, laser speckle imaging data were acquired with a frame rate of 60 Hz, and spatial frequency domain imaging data were acquired with a frame rate of 14 Hz. However, to reduce computational load, we reduced the sampling rate to ~ 17 data points per minute for spatial frequency domain imaging and ~ 39 data points per minute for laser speckle imaging, which was enough to capture the dynamic changes during SD.

### Data Analysis

#### Laser Speckle Imaging Data: Relative CBF

The laser speckle imaging data were used to compute the speckle flow index, a measure of CBF, over a fixed spatial region of interest spanning the majority of the imaging window. Relative cerebral blood flow (rCBF) was normalized to the mean CBF calculated over the 30-s interval immediately prior to the onset of asphyxia.

#### Spatial Frequency Domain Imaging Data: Tissue Scattering and Oxygenation

For the spatial frequency domain imaging data analysis, a custom MATLAB code [28] calculated best fit of a computational (Monte Carlo) model of light-tissue interaction to the measured data. These model fits provided tissue oxyhemoglobin concentration (ctHbO_2_), deoxygenated hemoglobin (ctHb), and scattering maps. The ctHbO_2_ and ctHb data were combined to calculate tissue oxygenation (StO_2_) via the following formula: StO_2_ = (100 × ctHbO_2_)/(ctHbO_2_ + ctHb). Time-resolved ctHbO_2_, ctHb, StO_2_, and tissue scattering curves were formulated by averaging the optical property maps over a fixed spatial region of interest.

#### Laser Speckle Imaging + Spatial Frequency Domain Imaging Data: CMRO_2_

Time-resolved CMRO_2_ was calculated using the formalism developed by Boas and Dunn [40, 42], combining CBF data from laser speckle imaging with ctHbO_2_ and ctHb data from spatial frequency domain imaging. For the calculation of CMRO_2_, the CBF data were analyzed using a region of interest covering the majority of the imaging window (to represent overall perfusion into the brain), whereas ctHbO2 and ctHb data were analyzed using an region of interest over a prominent vein (to more accurately probe the amount of oxygen consumed by the tissue).

#### Cerebrovascular Resistance Data

Cerebrovascular resistance (CVR) is the ratio of cerebral perfusion pressure to CBF. Relative cerebrovascular resistance versus time (rCVR[*t*]) was defined as rCVR(*t*) = relative mean arterial pressure versus time (rMAP[*t*])/relative cerebral blood flow versus time (rCBF[t]) [43–46], where rMAP and rCBF are values normalized to the mean over a 30-s interval immediately before onset of asphyxia.

#### Multimodal Parameter Set

For each cohort, a multimodal set of parameters was extracted from the data around SD onset to analyze several associations. In cohort 1, these parameters included the following: (1) time to DC-ECoG SD onset during asphyxial cardiac arrest for SD1 and SD2 and (2) average rate of change of MAP, systolic blood pressure, diastolic blood pressure, and heart rate 30 s before and after SD onset at both DC leads as well as between the SD onsets in the two leads (∆SD1-2 period).

In cohort 2, the parameter set included quantities obtained from the optical measurements. In this cohort, SD onset was most reliably measured via spatial propagation of changes in tissue scattering. The parameters extracted from cohort 2 included the following: (1) scattering change onset during asphyxial cardiac arrest (SD), (2) percentage change in scattering during SD, (3) area under the curve (AUC) of scattering change during SD, (4) time to peak of CVR, (5) maximum relative increase in CVR compared with baseline, and (6) AUC and slope over the 30 s before and after scattering change of MAP, systolic blood pressure, diastolic blood pressure, pulse pressure, and heart rate with the additional inclusion of CBF, CMRO_2_, CBF/CMRO_2_, and CVR. The ratio of CBF/CMRO_2_ is a metric of cerebral flow–metabolism (supply and demand) [27, 41].

#### Statistical Models and Tests

The personnel performing data analysis were not blinded to the experimental groups (cohort 1 and cohort 2). Statistical testing was performed with MATLAB. For each correlation analysis, Spearman correlations were performed when one of the parameters used was not normally distributed. When both parameters used for correlation were normally distributed, Pearson correlation was used. For each cohort, paired *t*-tests were performed to compare between time points for AUC and slopes.

## Results

### Detection of Anoxic SD During Asphyxial Cardiac Arrest with DC Electrodes

**Fig. 2:**
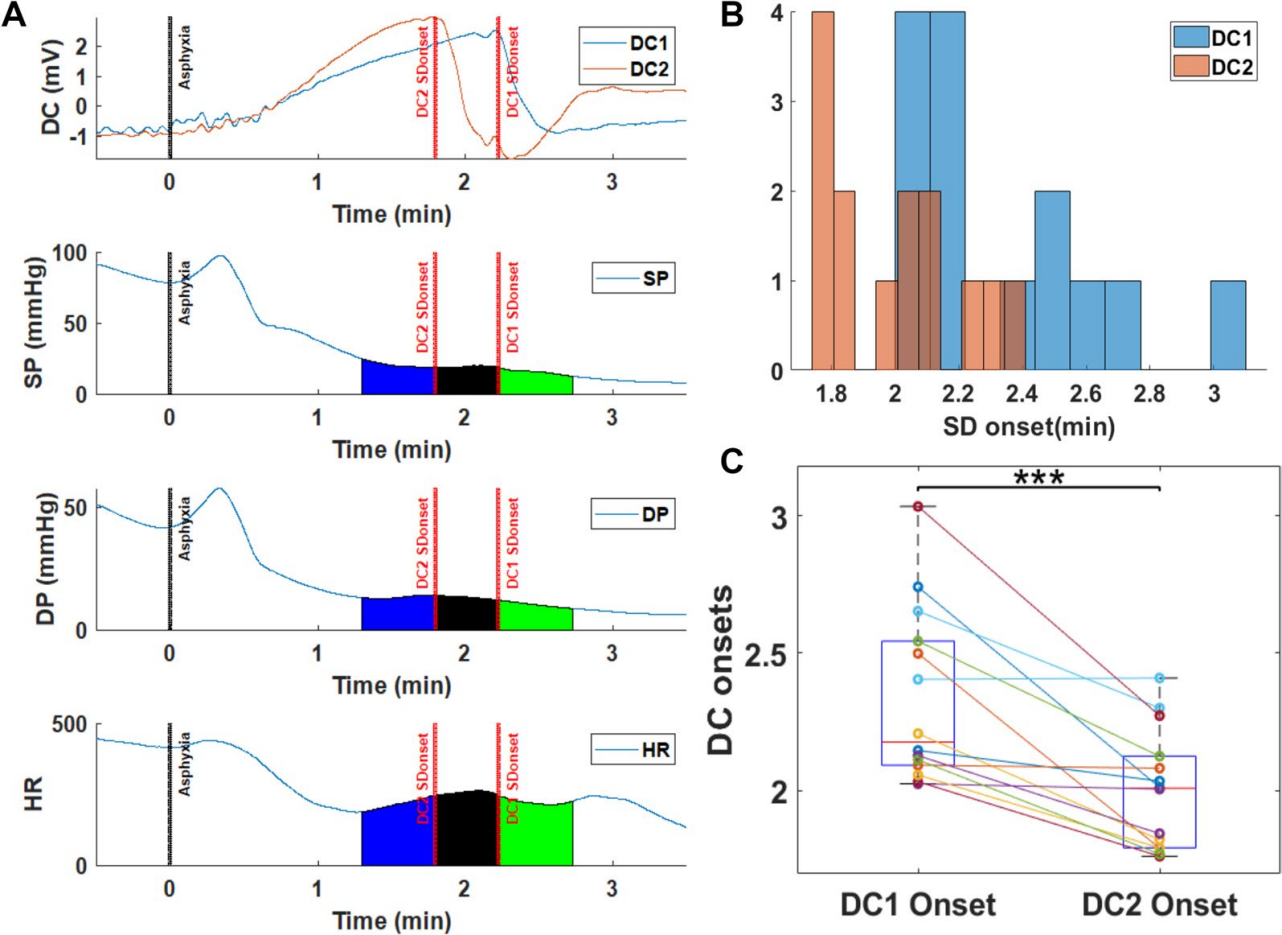
Detection of spreading depolarization (SD) during asphyxial cardiac arrest with DC electrodes in cohort 1. **a** Example DC potential tracing for rat undergoing asphyxial cardiac arrest, showing DC1 and DC2 potential shifts, as well as the corresponding systolic (SP) and diastolic (DP) blood pressures and heart rate (HR) during this period. **b** Distribution of SD onset times detected by DC1 and DC2 leads. SD occurs earlier in DC2 than DC1. **c** Paired one-tailed *t*-test showing that DC1 SD onset is significantly later than DC2 SD onset (*p < 0.05; **p < 0.01; ***p < 0.001; ****p < 0.0001)

In cohort 1, using DC electrodes, detection of SD occurred between 1.7 and 3 min after the onset of asphyxia in our cardiac arrest model (Fig. 2). For cohort 1, DC1 and DC2 were used to separately detect SD onset (Fig. 2), with DC2 detecting anoxic SD earlier than DC1. Because of this, the SD detected first by DC2 will be referred to as SD1, whereas the SD detected later by DC1 will be referred to as SD2. The SD was persistent until after successful resuscitation, at which point repolarization occurred. We refer to the period of delay in detection of anoxic SD between the two DC electrodes, located in different parts of the brain, as the ∆SD1-2 period. In the first part of this study, we analyzed the peripheral hemodynamic changes that occurred during this period of depolarizations of the brain.

### Diastolic Blood Pressure Drop Accelerates During ∆SD1-2 Period

**Fig. 3:**
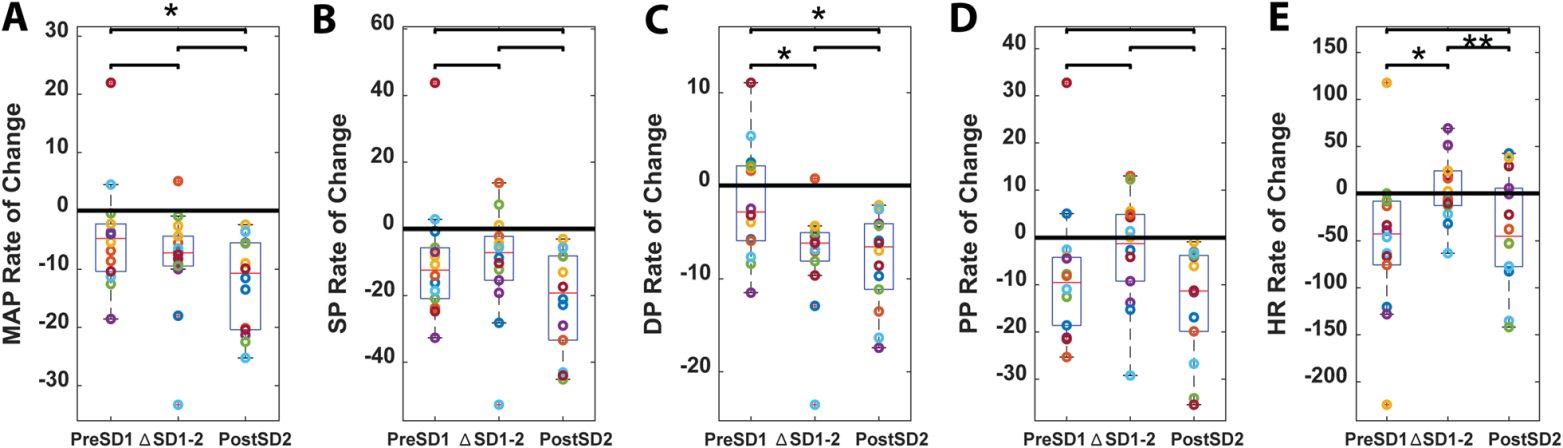
Diastolic blood pressure (BP) drop accelerates between SD1 and SD2 onsets. In cohort 1, rate of change for mean arterial pressure (MAP) (**a**), systolic BP (SP) (**b**), diastolic BP (DP) (**c**), pulse pressure (PP) (**d**), and heart rate (HR) (**e**) during the 30-s period before SD1 onset, the ∆SD1-2 period, and the 30-s period after SD2 onset. A negative rate of change value represents the drop rate. After SD1 onset, DP drop rate steepens, whereas SP drop rate remains stable and HR drop rate subsides and plateaus (*p < 0.05; **p < 0.01)

For cohort 1, MAP dropped faster in 30 s after the second detected SD compared with the MAP drop during the 30 s before the first detected SD (Fig. 3a). Systolic blood pressure and pulse pressure dropped at a consistent rate because the rate of change was not significantly different between the three time periods (Figs. 3b, c). Of note, in contrast to other blood pressures, diastolic blood pressure dropped faster within the same time frame (Fig. 3c). Figure 3e shows that the rate of change of the heart rate curve actually plateaus during the ∆SD1-2 period. This indicates that heart rate was dropping before it stabilized and was maintained during the ∆SD1-2 period then dropped again afterward.

### Peripheral Blood Pressure is Transiently Decoupled from Heart Rate During SD

**Fig. 4:**
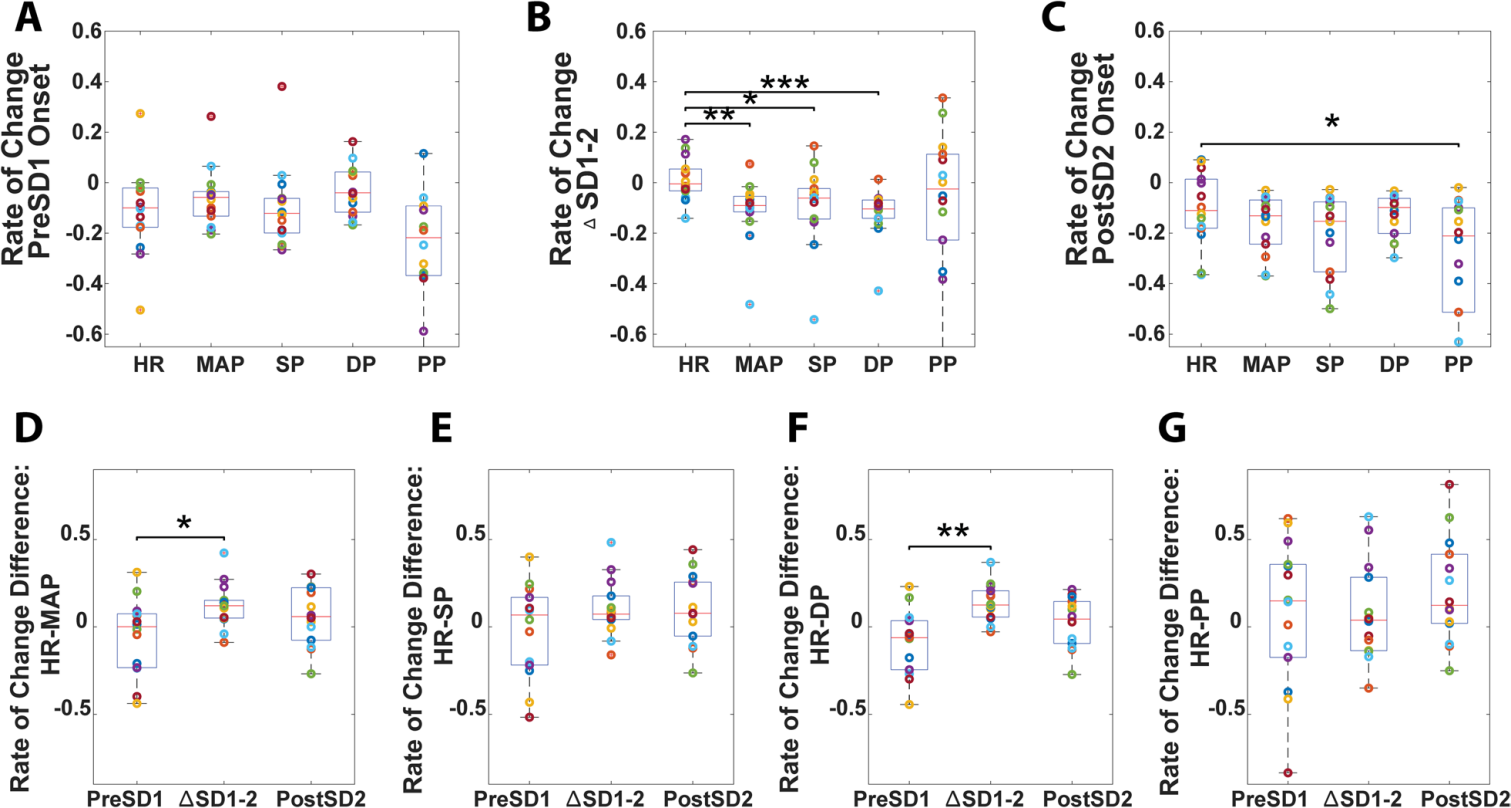
Heart rate (HR) and blood pressure (BP) become transiently decoupled during SD and partially recoupled after spreading depolarization (SD). **a** In cohort 1, average rates of change (slope) during the 30-s period before SD1 (pre-SD1) of HR, mean arterial pressure (MAP), systolic BP (SP), diastolic BP (DP), and pulse pressure (PP). Prior to calculating rates of change, each of these parameters was normalized to baseline so that the parameters could be compared with each other. There is no difference in the rate of change between HR and BP before SD1 onset. **b** HR and BP metrics (as in **a**) during the ∆SD1-2 period. During SD, there is a significant difference between the average rate of change of HR and BP parameters. **c** HR and BP metrics during the 30 s after SD2 (post-SD2). Difference in rate of change (slope) between HR and MAP (**d**), SP (**e**), DP (**f**), and PP (**g**): pre-SD1, ∆SD1-2, and post-SD2. HR relative to MAP and DP shows significant initial decoupling with a recoupling post-SD2, whereas, relative to SP and PP, HR shows no significant decoupling throughout (*p < 0.05; **p < 0.01)

Heart rate and blood pressure were closely associated physiologically. Using values normalized to baseline (30 s before asphyxia), rate of change (slope) in heart rate was compared with rate of change in normalized blood pressure (Fig. 4) during the same time periods as in Fig. 3: pre-SD1, ∆SD1-2 period, and post-SD2 (Fig. 4a–c, respectively). The values were normalized to obtain relative (percentage) changes so that heart rate and blood pressure could be compared individually and between each other because in a clinical setting, percentage changes of these values are important for assessing disorders, such as heart failure. We saw that rate of change in heart rate was well coupled to rate of change in all peripheral blood pressure measures prior to the first SD onset as expected (Fig. 4a). However, heart rate increased and/or plateaued during the ∆SD1-2 period, whereas blood pressure continued to drop (Fig. 4b). Thus, the rates of heart rate and blood pressure drop became different, indicating decoupling of the heart rate and blood pressure during this period. Figure 4c shows that after detection of the second SD onset, the heart rate began to fall again and partially closed the gap with the drop rates of the blood pressures. After detection of the second SD onset, heart rate again became coupled with blood pressure, with the exception of pulse pressure. Figure 4d shows the difference in rate of change of relative heart rate and MAP (heart rate–MAP) during the same time points. The difference between heart rate and MAP being close to zero before the first SD onset indicates how well coupled they began prior to their significant decoupling during the ∆SD1-2 period. They became coupled once again after the second SD onset as the change became nonsignificant in relation to before the first SD onset (*p* > 0.05). A similar trend is shown with diastolic blood pressure in Fig. 4f. However, a significant difference in coupling of heart rate with systolic blood pressure and pulse pressure was not seen (Fig. 4e, g).

### Shorter ∆SD1-2 Period and Faster Drop in MAP are Associated with Better Neurological Outcome Post Cardiac Arrest

**Fig. 5:**
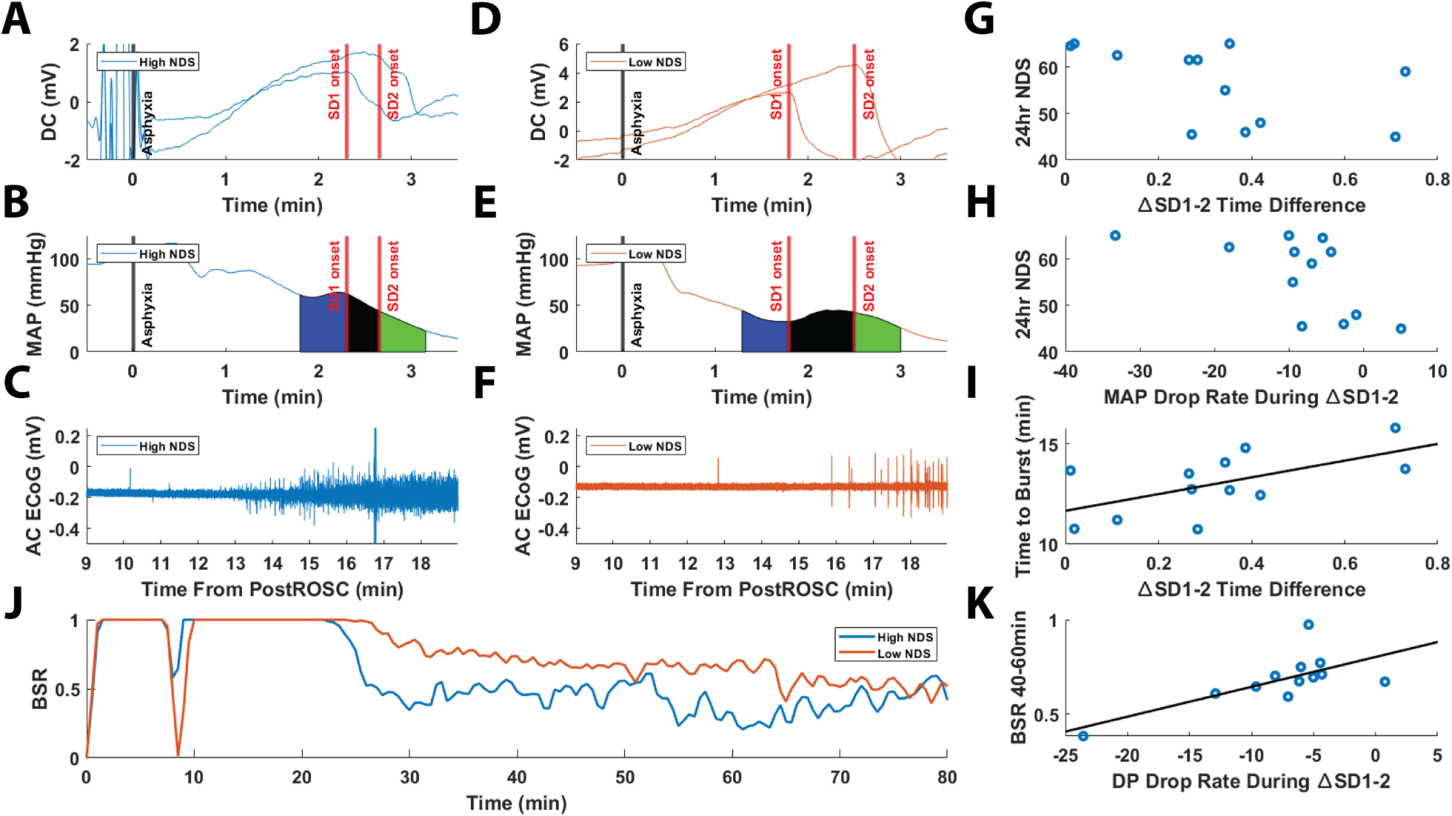
Shorter ∆SD1-2 period is associated with better neurological outcome post cardiac arrest. In cohort 1, spreading depolarization (SD) onsets (**a**) mean arterial pressure (MAP) (**b**), and AC ECoG (**c**) for an animal with good outcome (high 24-h neurological deficit scale [NDS] score). SD onsets (**d**), MAP (**e**), and AC ECoG (**f**) for an animal with a poor outcome (low 24 h NDS score). **c** Shows an earlier time to burst (TTB), defined as the resumption in cerebroelectrical activity, in the AC ECoG signal post resuscitation in a rat with good outcome, whereas **f** shows a later TTB in a rat with poor outcome. **j** shows corresponding rats from **c** and **f** with their burst suppression ratios (BSRs), with the good outcome rat (**c**, blue tracing) having a lower BSR and poor outcome rat (**f**, orange tracing) having a high BSR. **g** shows that a shorter ∆SD1-2 period is associated with a higher 24-h NDS score (Spearman *p* < 0.05, *r* =  − 0.60). **h** shows that a faster drop rate in perfusion (as measured by slope of MAP) during the ∆SD1-2 period is associated with a higher 24-h NDS score (Spearman *p* < 0.05, *r* =  − 0.69). **i** shows that a shorter ∆SD1-2 period is correlated with shorter TTB (Pearson *p* < 0.05, *r* = 0.60). **k** shows that a faster drop rate of (diastolic blood pressure (DP) during the DC2-DC1 SD onset time period is associated with less burst suppression at 40 to 60 min post resuscitation, indicating a better outcome (Pearson *p* < 0.005, *r* =  − 0.77)

The rats with a good outcome (blue tracing, Fig. 5a) were seen with a shorter ∆SD1-2 period, a faster drop rate in MAP during the same time period (Fig. 5h), an earlier time to first burst or time to burst (Fig. 5c), and a lower burst suppression ratio (Fig. 5j). The rats with poor outcome were seen to have a longer ∆SD1-2 period, slower MAP drop rate during that period, later time to burst, and higher burst suppression ratio (Fig. 5d, e, f, j, respectively). Mean perfusion varied from rat to rat during the ∆SD1-2 period (mean MAP 21 ± 10 mm Hg, range 7–40 mm Hg). The mean MAPs for pre-SD1 and post-SD2 were 23.05 ± 9.07 mm Hg and 16.58 ± 8.45 mm Hg, respectively. The mean values at any of these periods did not correlate with any of our outcome measures. The same was found for diastolic blood pressure, systolic blood pressure, and pulse pressure (not shown). However, we found that a faster drop in MAP during this period was associated with better neurological outcome as measured by the behavioral neurological deficit score 24 h post resuscitation (Fig. 5h). We also found that a faster drop rate of diastolic blood pressure during the ∆SD1-2 period was associated with faster recovery of electrical brain activity, indicated by a lower burst suppression ratio or, more simply, an increase in ECoG bursting 40 to 60 min post resuscitation (Fig. 5k). Lastly, we found that a shorter ∆SD1-2 period was correlated with a better 24-h neurological deficit score (Fig. 5g) as well as an earlier resumption of electrical activity in the brain, measured by time to burst (Fig. 5i). Clinically, this means that a shorter duration of ∆SD1-2 in the brain and a faster drop rate of peripheral blood pressure during SD in global ischemia are associated with better outcome. This was only done for cohort 1 because the rats in cohort 2 were euthanized ~ 90 min after resuscitation.

### Magnitude of SD-Dependent Changes in Tissue Scattering Associated with Subsequent Changes in CVR

**Fig. 6:**
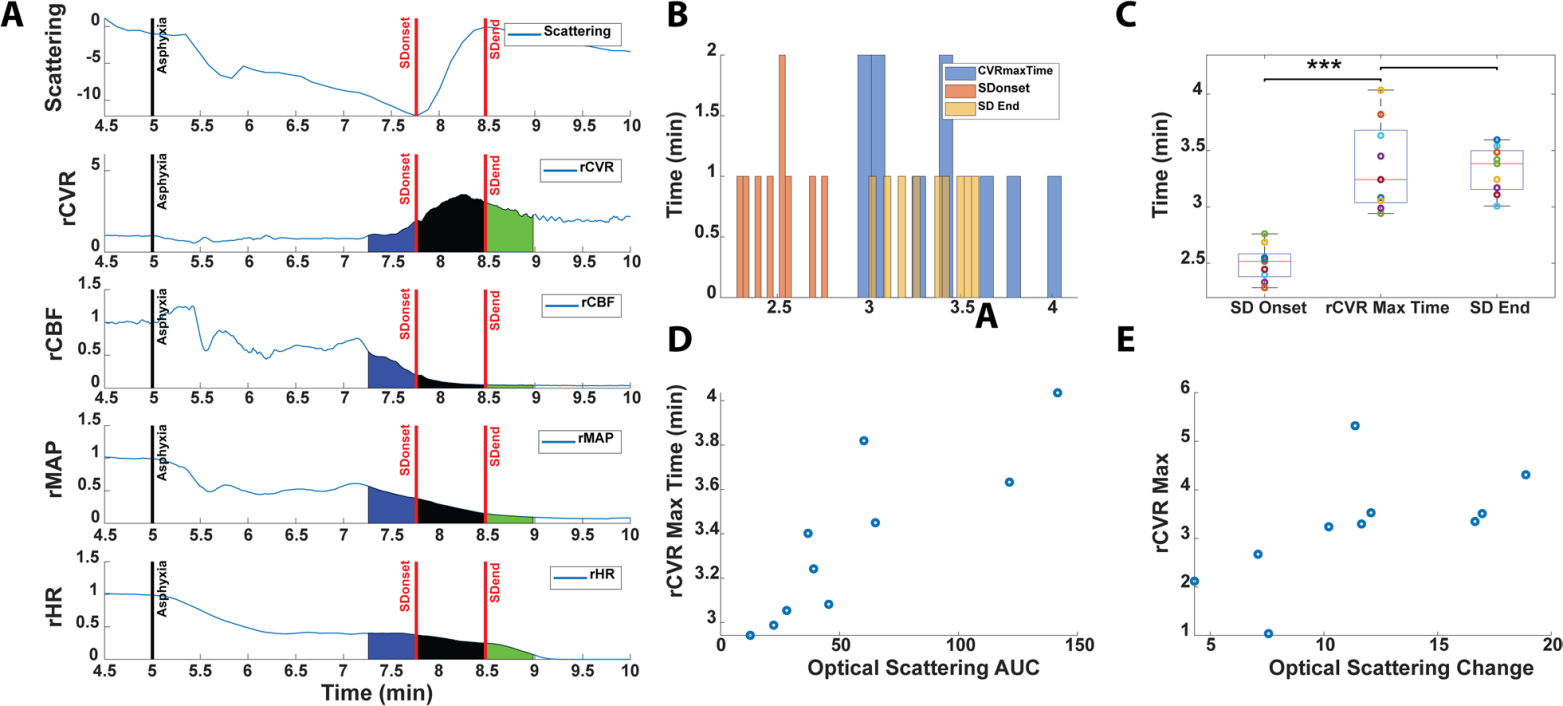
Spreading depolarization (SD), measured by optical scattering, is associated with changes in cerebrovascular resistance (CVR). **a** In cohort 2, tracings of tissue scattering changes in the brain, along with relative cerebrovascular resistance (rCVR), relative cerebral blood flow (rCBF), relative mean arterial pressure (rMAP), and relative heart rate (rHR), in a representative rat, all normalized to baseline. **b** Distributions of SD onset time, completion time, and time at which CVR peaks in cohort 2 animals. **c** Comparison of SD onset and SD end times vs. rCVR maximum time, showing that rCVR maximum time occurs significantly after SD onset and occurs around SD end time (***p < 0.0005). **d** Scatter plot showing positive correlation between optical scattering area under the curve (AUC) during SD and rCVR maximum time (Spearman *p* < 0.0005, *r* = 0.92). **e,** Scatter plot showing positive correlation between magnitude of optical scattering change during SD and rCVR peak value (Spearman *p* < 0.05, *r* = 0.75)

In cohort 1, we analyzed how SD may affect peripheral dynamics but did not measure any cerebral hemodynamic changes. In cohort 2, we measured the cerebral hemodynamic changes in concert with peripheral hemodynamic changes during SD to determine whether SD affected both the same way. SD in cohort 2 was monitored with optical imaging and was measured via optical scattering changes in the brain (Fig. 6a). Owing to the optical window taking the place of where DC2 lead would go, we could not analyze the exact same duration of SD using the two DC leads as in cohort 1. Also, because we observed scattering change through one cranial window, we were unable to spatially discern if the scattering changes were multiple or a single wave. The wave of scattering change we observed may be a composition of multiple waves, but it is unclear. Although a different metric of SD from cohort 1, this still provided cerebral hemodynamic changes to be compared with peripheral hemodynamic changes that occurred during SD. The onset of the scattering change (defined as the local minimum point on the scattering curve) was marked as the onset of SD. The maximum point at the end of the scattering change was marked as the end of SD. This period from the onset to the end simply indicates the time in which we observed scattering change in our window and likely does not represent the state of the entire brain. The changes in rCBF, rMAP, and rHR (Fig. 6a) were more subtle during the scattering change (SD) compared with before and after SD, but the rise and peak in rCVR was apparent during this period. The peak rCVR occurred near the end of the SD-related optical scattering change (Fig. 6b, c). The time at which rCVR peaked was also strongly correlated with the AUC of optical scattering change (Fig. 6d). The peak value of rCVR during this period correlated with the magnitude of optical scattering change (Fig. 6e). These findings indicate that the timing and magnitude of SD, as measured by optical scattering, is correlated to changes in CVR.

### Significant Transient Rise of CVR Coincides with Perturbation in Cerebral Flow–Metabolism Coupling During SD

**Fig. 7:**
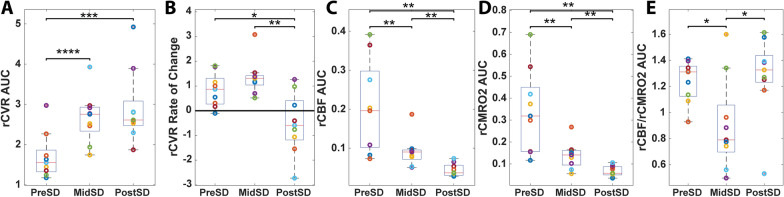
Rise in cerebrovascular resistance (CVR) coincides with perturbed flow–metabolism coupling. **a** In cohort 2, area under the curve (AUC) of relative cerebrovascular resistance (rCVR) normalized to the 30-s period before spreading depolarization (SD) (pre-SD), during the span of SD (mid-SD) (duration of scattering change), and the 30-s period after SD (post-SD). Compared with before SD, there is a significant increase in the CVR mid-SD and post-SD. **b** Rate of change of rCVR through SD time points. The rate of change of CVR increases mid-SD compared with pre-SD but decreases significantly post-SD. **c** AUC of relative cerebral blood flow (rCBF) through SD time points. rCBF dramatically drops from mid-SD onward. **d** AUC of relative cerebral metabolic rate of oxygen (rCMRO_2_) through SD time points. rCMRO_2_ drops dramatically mid-SD and post-SD. **e** rCBF/rCMRO_2_ is a metric of cerebral flow–metabolism (e.g., supply and demand). Although the individual AUCs of both rCBF and rCMRO_2_ decrease mid-SD and post-SD, the rCBF/rCMRO_2_ AUC ratio normalized to time shows significant reduction during mid-SD only

With the onset of SD, there was a rise in CVR that persisted through SD and after (Fig. 7a). However, with the end of SD, the rate of change of rCVR became negative, indicating a decrease in CVR (Fig. 7b). rCBF and rCMRO_2_ (with ‘r’ indicating values relative to baseline) consistently dropped throughout the course of SD (Figs. 7c, d). However, there was a noticeable SD-related change in the rCBF/rCMRO_2_ ratio, which is a metric of cerebral flow–metabolism (supply and demand) [27, 41]. The AUC of the rCBF/rCMRO_2_ ratio (AUC rCBF/AUC rCMRO_2_ then normalized to duration) transiently but significantly dropped during SD, indicating cerebral flow–metabolism decoupling, but returned to pre-SD values after SD (Fig. 7e).

### After SD, the Drop in Peripheral Blood Pressure is Decoupled from Cerebral Perfusion and Metabolism

**Fig. 8:**
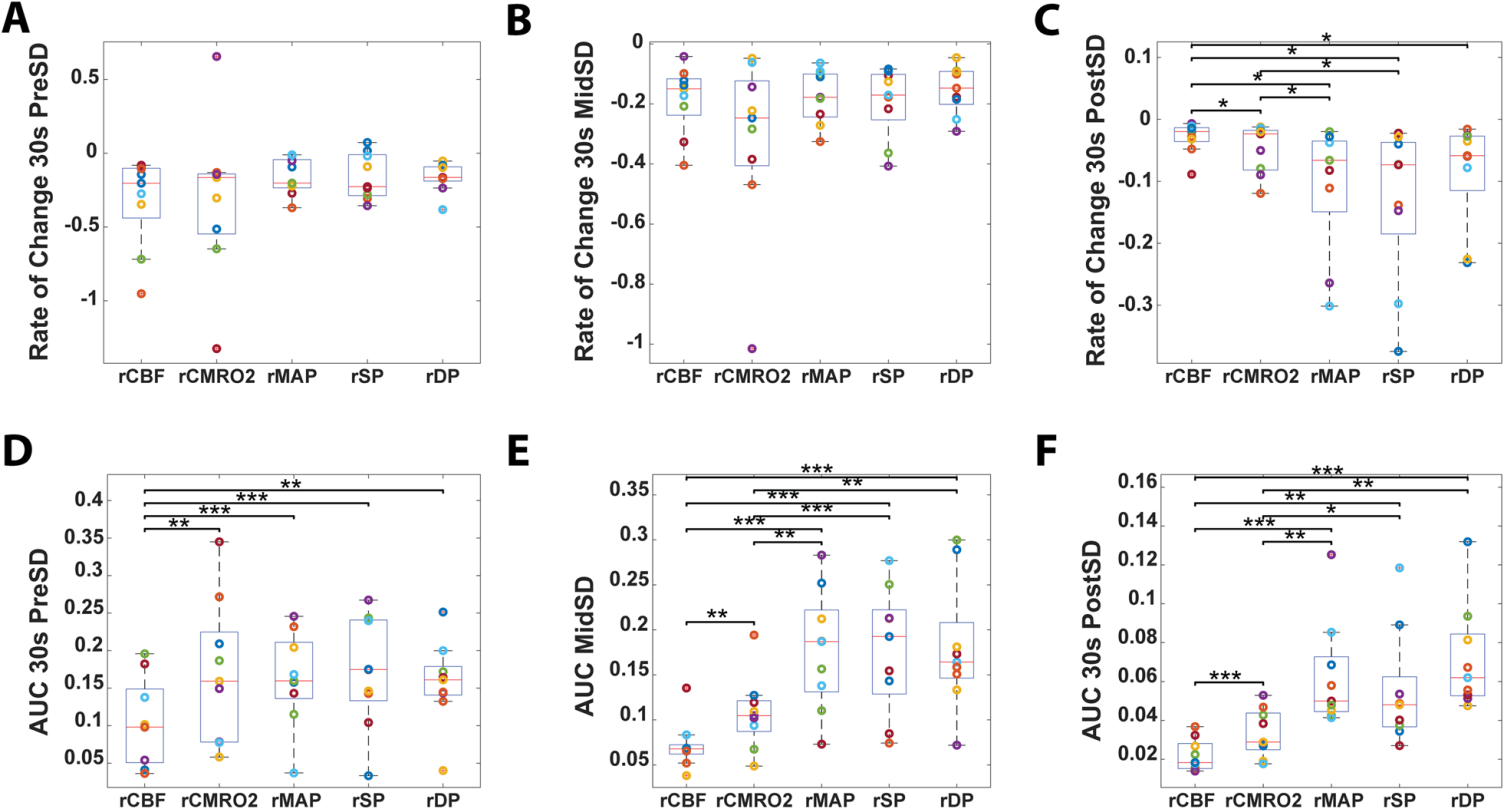
Decoupling of peripheral blood pressure (BP) from cerebral perfusion and metabolism mid-spreading depolarization (mid-SD) and/or post-SD. In cohort 2, the rate of change of relative cerebral blood flow (rCBF), relative cerebral metabolic rate of oxygen (rCMRO_2_), relative mean arterial pressure (rMAP), relative systolic BP (rSP), and relative diastolic BP (rDP) pre-SD (**a**), mid-SD (**b**), and post-SD (**c**). There is no difference between rCBF and other parameters pre-SD and mid-SD, but there is a significant difference post-SD. Area under the curve (AUC) of rCBF, rCMRO_2_, and peripheral BPs pre-SD (**d**), mid-SD (**e**), and post-SD (**f**). rCBF is significantly lower than the other parameters pre-SD, mid-SD, and post-SD. rCMRO_2_ is only significantly different from peripheral BP mid-SD and post-SD

Pre-SD and mid-SD, the rates of change in CBF, CMRO_2_, and peripheral blood pressure were similar (Fig. 8a, b), signifying coupling between these parameters. Post-SD, CBF became decoupled to both CMRO_2_ and peripheral blood pressure (Fig. 8c). During this period, the rate of drop in CBF was closer to zero than that in CMRO_2_ and peripheral blood pressure. This was likely due to the maximum CVR occurring at the same time, shutting off blood flow and causing decoupling between CBF and peripheral blood pressure. Similarly, CMRO_2_ was coupled with peripheral blood pressures pre-SD (Fig. 8d) but became decoupled from peripheral blood pressures at the start of SD (Fig. 8e, f). CMRO_2_ dropped dramatically with SD onset, much greater than the relative drops in peripheral blood pressures (Fig. 8e, f). However, CBF AUC was significantly lower than CMRO_2_ AUC and peripheral blood pressure AUC throughout all time points.

## Discussion

In this study, we investigated the effects of anoxic SD on CBF, cerebral metabolism, and peripheral blood pressure during global ischemia and its effect on neurological outcome after resuscitation. Although most literature focuses on the effect of SD on local cerebral perfusion or the effect of ischemia/hypotension on induction of anoxic SD, here we focused on the reverse possibility: the potential for anoxic SD to invoke changes in peripheral blood pressure. Moreover, to our knowledge, this is the first study to characterize the relationship between peripheral blood pressure and CBF during anoxic SD. A previous study [18] using multimodal imaging of anoxic depolarization used voltage-sensing dye to measure SD and investigated CBF with laser speckle imaging and peripheral blood pressure during SD in a nonresuscitation cardiac arrest model, but the analysis concluded that SD did not affect either CBF or peripheral blood pressure. However, the authors of the aforementioned study did not assess the data with a high temporal resolution during SD because they only used averaged signals at seven selected time points over 3 min, which was too low to capture rapid changes [18]. By contrast, our study measured ~ 17 data points per minute with spatial frequency domain imaging and ~ 39 data points per second with laser speckle imaging. Additionally, the previous study did not characterize the relationship between CBF and peripheral blood pressure, but rather it looked at them independently, finding that anoxic SD did not affect the changes in CBF or peripheral blood pressure. In contrast, we found that anoxic SD may affect peripheral blood pressure, possibly by relaxing peripheral vasculature, and decouple peripheral blood pressure from CBF and metabolism. Furthermore, in our resuscitation cardiac arrest model, we show for the first time an association between the characteristics of peripheral blood pressure during anoxic SD and neurological outcome.

In cohort 1 animals, the ∆SD1-2 period, a time delay between detection of two anoxic SDs measured in two different locations, was analyzed. We found this period to be significant because we found the time delay varied from rat to rat, and it may represent a broad connectivity metric across the brain while it is shutting down. Furthermore, we noticed blood pressure fluctuations during this period, and so we investigated the potential peripheral and cerebral changes occurring during this period. As mentioned in the Results section, we cannot be certain that the detected SDs were from separate foci or from a single propagating SD, but it is likely more than one anoxic SD on the basis of the global ischemia model [25]) and that the speed of propagation (if a single SD) would be much too fast compared with what is known in literature. Using the distance between the two DC electrodes and the time delay between the two, the calculated speed of the anoxic SD ranged from 7 to 724 mm/min. On the basis of known SD speeds of 1.5 to 9.5 mm/min, these speeds were too fast, which suggests that rather than a single propagating wave, there were likely multiple foci where SD originates, leading to, in some cases, near simultaneous detection at both DC electrodes. This may be because in this asphyxial cardiac arrest model, blood flow to the whole brain shuts down, leading not to a single, but likely multiple ischemic cores occurring at near simultaneous times because of differences in ischemic thresholds in different regions of the brain based on possible proximity to blood vessels and local blood flow and metabolic demand. Per prior studies, during global ischemia, anoxic SD is likely to occur at slightly different times in different brain regions, possibly reflecting small variations in tissue perfusion loss during ongoing ischemia and/or different regions having varying thresholds for triggering of anoxic SD [47–49]. In contrast, in focal stroke or other conditions in which there is a single ischemic core, the SD propagates outward from the ischemic core.

### Cortical Anoxic SD Transiently Decouples Peripheral Blood Pressure from Heart Rate

Effects of SD on cerebral hemodynamics and metabolism have been investigated [2], but the effects of SD on peripheral blood pressure and heart rate remain largely unexplored. Our data showed that the rate of change of systolic blood pressure was consistent from before the ∆SD1-2 period, during the ∆SD1-2 period, to after, whereas diastolic blood pressure’s drop rate increased throughout the same time periods. This contrasts with the transient rise and/or plateauing of heart rate we observed during the ∆SD1-2 period, which was accompanied by stronger systolic function that prevented the systolic blood pressure from dropping at an accelerated rate. Although these may appear to be compensatory cardiac functions in a state of circulatory shock, the diastolic blood pressure drop rate continued to accelerate, supporting a possible relaxation of the peripheral vascular tone and/or changes in cardiac diastolic function. Because diastolic blood pressure is closely associated with vascular tone and elasticity [50, 51] more than cardiac function, it is possible that anoxic SD may be indirectly affecting peripheral vascular tone. Future studies will focus on direct assessment of peripheral arteries to assess for vasodilation and evaluation of cardiac function for changes in diastole. This is further supported by the changes in heart rate being coupled with changes in peripheral blood pressure before the first SD onset, whereas during the ∆SD1-2 period, the heart rate transiently rose, decoupling it from all the peripheral blood pressure metrics, and after detection of the second SD onset, became less decoupled. These data suggest that anoxic SD may possibly produce a transient brain–heart coupling effect, which increases heart rate while relaxing vascular tone. One mechanism of such brain–heart coupling may be via the vagus nerve, which can modulate the parasympathetic activity of the heart, including regulation of both the sinoatrial nodes and atrioventricular nodes [52]. Interestingly, the vagus nerve has recently been shown to modulate SD [11–14]; however our data suggest that the reverse directionality is plausible, namely that anoxic SD may also potentially modulate the heart through the vagus nerve or its related components.

### Shorter ∆SD1-2 Duration and Faster Drop in Peripheral Perfusion During the ∆SD1-2 Period is Associated with Better Outcome 24 Hours Post Cardiac Arrest

We found that a faster drop in peripheral perfusion (slope of MAP and diastolic blood pressure) during the ∆SD1-2 period was correlated with a higher behavioral recovery 24 h later (neurological deficit scale scores) (Fig. 5h). This suggests that faster drop in peripheral perfusion during the ∆SD1-2 period is associated with better neurological outcome. However, we also showed that a shorter ∆SD1-2 period was also associated with better outcome, as evidenced by shorter time to burst (Fig. 5i), as well as increased behavioral recovery 24 h later (Fig. 5g). We also saw that a faster drop in diastolic blood pressure during the ∆SD1-2 period was associated with better neurological outcome in quantitative ECoG parameters immediately post resuscitation and better neurological outcome post cardiac arrest (Fig. 5k). This may appear counterintuitive because faster depression of the brain with anoxic SD and a faster drop in perfusion may be expected to produce worse outcomes. However, in another recent study from our research laboratory, we again found that a faster drop in perfusion before anoxic SD is associated with better neurological outcomes after cardiac arrest [9]. It is possible that there may be an unmeasured component related to SD and perfusion that produces a protective effect. Future studies will focus on teasing these complex mechanisms apart.

### Magnitude of Anoxic SD is Associated with Subsequent Changes in CVR and Transient Decoupling of CBF and Metabolism During Anoxic SD

Our multimodal platform using optical imaging of the brain alongside peripheral blood pressure monitoring allowed us to simultaneously and continuously monitor CBF, brain metabolism, and peripheral blood pressure throughout the cardiac arrest experiment. Using the measured CBF and MAP, we estimated a surrogate measure of rCVR [43–46]. We suspected that rCVR may be particularly sensitive to changes in CBF and blood pressure during anoxic SD given that SD is known to affect CBF and our data in this study support blood pressure changing after anoxic SD onset. Interestingly, we observed a significant increase in rCVR starting with the onset of SD-associated changes in optical scattering. During SD, there were morphological changes in tissue that increased scattering of light, which could be detected by spatial frequency domain imaging and used to determine the onset of SD [9]. Under normoxic conditions, SD is known to induce vasodilation to increase CBF [2], but in pathological conditions (e.g., ischemic or hypoxic), it has been shown to reduce vessel diameter [18], thus increasing CVR. However, to our knowledge, the changes that occur in peripheral and cerebral hemodynamics as well as cerebral metabolism 30 s before, during, and 30 s after anoxic SD have not been analyzed with high temporal resolution. High temporal resolution was needed because it allowed us to separate the dynamics between these 30- and 60-s periods to provide insights into the physiology of cerebral and peripheral hemodynamics before, during, and after anoxic SD. In addition to the rise of CVR with anoxic SD onset, we also found that the peak of CVR coincided with the end of optical scattering changes (end of anoxic SD). After anoxic SD, CVR interestingly began to decrease. The time at which CVR peaked was also strongly correlated with the AUC (a measure of total magnitude) of optical scattering change during anoxic SD (*p* < 0.0005, *r* = 0.92). Additionally, peak CVR was also strongly correlated with the magnitude of optical scattering change during SD (*p* < 0.05, *r* = 0.75). Greater observed optical scattering changes may be related to anoxic SD-induced extracellular ion changes (i.e., increase K^+^ ions) and thus greater vasoconstriction. This aligns with findings from another study in which extracellular K^+^ was the predominant vasoconstrictor in SD [2, 53]. This may explain how CVR peaks at the end of anoxic SD and how the magnitude of anoxic SD or extracellular K^+^ ion change is correlated with the magnitude of CVR. Moreover, whereas immediately before, during, and after anoxic SD, CBF and CMRO_2_ continuously drop, the ratio of rCBF/rCMRO_2_, a metric of cerebral flow–metabolism coupling, showed a different trend. Specifically, during anoxic SD, rCBF/rCMRO_2_ dramatically dropped but then returned to its previous state after anoxic SD. We speculate that the rise in CVR was caused by CBF decreasing more than CMRO_2_, thus producing the flow–metabolism imbalance during anoxic SD.

### Anoxic SD is Associated with Decoupling of Peripheral Blood Pressure from CBF and Brain Metabolism

Before and during anoxic SD, the rate of change in CBF was coupled with that of CMRO_2_ and peripheral blood pressure, suggesting that the changes in all these parameters are similar. After anoxic SD, CBF became decoupled from rCMRO_2_ and peripheral blood pressure. During this period, the rate of drop in CBF was closer to zero compared with that in CMRO_2_ and peripheral blood pressures, indicating that it had likely fallen to a minimum and could not be reduced further. This is likely due to CVR hitting its peak at this time, nearly shutting down blood flow and causing decoupling of CBF from CMRO_2_ and peripheral blood pressure. Similarly, CMRO_2_ was coupled with peripheral blood pressures before anoxic SD but became decoupled starting at anoxic SD onset. CMRO_2_ dropped dramatically with anoxic SD onset with a magnitude much greater than that of the relative drops in peripheral blood pressures. This may be because anoxic SD is a highly energy-consuming event, and with a lack of oxygen to begin with, cerebral metabolism consumes any remaining oxygen and shuts down quicker. This is in contrast to SD in normal condition with sufficient oxygen, in which CMRO_2_ increases to the point of potentially doubling compared with baseline. These data further show that unlike in a normal state, during an ischemic-hypoxic state, anoxic SD leads to a dramatic relatively greater drop in both CBF and CMRO_2_, leading to a decoupling of both with peripheral blood pressure during and after anoxic SD.

## Limitations

Our study has several limitations that we hope to address in future experiments. A major limitation in our study is that some of our findings and relationships between factors are correlational. Future studies will require interventions during ongoing global ischemia to better assess causality and the specific mechanisms that may tie anoxic SD with cardiovascular function and neurological outcome. Along these lines, this report does not characterize cerebral changes and neurological recovery data on a molecular, cellular, and histological level, which would help to better elucidate the mechanisms underlying our findings. In addition, our sample size was low and because of the high number of statistical comparisons, there is a concern for underpowering and type II error in this pilot study. The uncoupling effects seen in our results may also simply be due to cardiac dysfunction occurring at the same time as SD. It is unclear whether the cerebral dysfunction or cardiac dysfunction played a greater role, but because of some of the hemodynamic effects being transient just during SD, it seems less likely it is purely due to cardiac dysfunction. Furthermore, we only had two DC electrodes and we also did not test whether these results would hold true if the electrodes were to be placed in different regions of the brain. Future experiments can include more DC electrodes and in different locations to test if the results still hold and to be able to better decipher the pattern of connectivity with greater spatial resolution. Another major limitation is that this study only used male animals. Sex differences are critical to a variety of neurological and cardiovascular functions, and future experiments should account for this by including both sexes. Furthermore, animals were divided into two cohorts with different surgical preparations, precluding us from obtaining behavioral outcome data (e.g., neurological deficit scale score) 24 h later for animals in the optical imaging cohort because they were euthanized after 2 h of monitoring following resuscitation due to the craniectomy. Additionally, a craniectomy can introduce its own confounding variable because it provides an outlet for intracranial pressure changes that may occur during ischemia. Although this is a limitation, it also may indeed make our rCVR estimation more reliable because a craniectomy helps to mitigate major changes in intracranial pressure [43–46]. The craniectomy also may affect CBF. Nevertheless, limitations with a craniectomy can be addressed in future studies by using either a chronic imaging window or a thinned skull procedure to enable survival studies out to 1 week post resuscitation or beyond. Finally, the craniectomy was performed over only a 6 × 4 mm area of the skull rather than the entire surface of the brain. Therefore, our measurements of SD onset in cohort 2 were limited to the location and time the CBF and scattering changes first appeared within the craniectomy region. Another limitation of this study is that we did not measure partial pressure of CO_2_ in the blood or local pH continuously throughout asphyxia and anoxic SD, which may affect anoxic SD.

## Conclusions

In this study, we used a rat model of asphyxial cardiac arrest with continuous and simultaneous multimodal monitoring, including optical imaging and electrophysiology, to quantify changes in cerebral activity, blood flow, oxygen metabolism, peripheral blood pressure, and heart rate before, during, and after anoxic SDs. We analyzed the effects of different stages of anoxic SD on CBF, CMRO_2_, and peripheral blood pressure as well as the relationship between these three metrics. Although the effect of hypoperfusion on anoxic SD has long been known, our study demonstrates for the first time that anoxic SD may in turn have effects on peripheral perfusion not only by affecting CBF and CMRO_2_ but also by stretching further, leading to changes in the peripheral blood pressure measured in the extremities and heart rate likely via a brain–heart connection. We show that there is a significant transient rise of CVR during anoxic SD that is positively correlated with the magnitude of anoxic SD and that the rise in this CVR coincides with perturbation in cerebral flow–metabolism coupling during anoxic SD. We also show that a faster drop of peripheral perfusion during anoxic SD is associated with better short-term (40–60 min) and longer-term (24 h) neurological recovery after cardiac arrest. These results provide support for the likelihood that the influence of anoxic SD may not be limited to the brain but can likely reach the peripheral cardiovascular system. Peripheral blood pressure is generally monitored in a critical care setting in the hospital, so characterizing the effects of anoxic SD on peripheral blood pressure may have significant potential for clinical translation and potential interventions. Further investigation into the relationship between the peripheral cardiovascular system and the brain during anoxic SD may provide new insights into understanding a potentially critical role for anoxic SD in global ischemia and recovery [9].
